# Can Subacute Thyroiditis Be a Cause of Fever of Unknown Origin?

**DOI:** 10.7759/cureus.16399

**Published:** 2021-07-15

**Authors:** Bhagyalakshmi Satyanarayan, Anupama Sahu, Satish K Prasad, Sarita Kumari

**Affiliations:** 1 Internal Medicine, Tata Main Hospital, Jamshedpur, IND

**Keywords:** upper respiratory tract infections, subacute thyroiditis, pyrexia of unknown origin, nonsteroidal anti-inflammatory drugs, glucocorticoids

## Abstract

Subacute thyroiditis (SAT) is a self-limiting, painful, non-suppurative thyroid gland inflammation, which usually develops two to eight weeks after viral upper respiratory tract infections, accompanied by pain and fever. The thyroid gland is large, painful, and tender. It presents with fever, myalgia, arthralgia, weakness, and sore throat.

A 37-year-old male presented to medical ward with a history of fever for three weeks along with swelling of neck and difficulty in swallowing. The patient had features of hyperthyroidism. High-resolution ultrasonography (HRUSG) and thyroid scan were suggestive of thyroiditis. Later on, on follow-up, the patient developed hypothyroidism. He was managed with antibiotics, nonsteroidal anti-inflammatory drugs (NSAIDs), and glucocorticoids, and he responded well to the above treatment. The index patient presented as a case of fever which on workup was found to be secondary to subacute thyroiditis (SAT).

## Introduction

Subacute thyroiditis (SAT) also known as giant cell, granulomatous, painful, or de Quervain's thyroiditis [1,2] is a self-limiting, non-suppurative thyroid gland inflammation, which usually develops after viral upper respiratory tract infection, accompanied by fever and pain in the area of thyroid gland but can also uncommonly present as painless thyroid gland and fever [3]. Possible causes of thyroiditis include viruses like mumps, measles, influenza, Epstein Barr virus, human immunodeficiency virus, and adenoviruses, hepatitis B, cytomegalovirus, enterovirus, coxsackie viruses A and B [4] and now also severe acute respiratory syndrome coronavirus 2 (SARS-CoV-2) [3,5]. However, the exact etiology of SAT is unknown. Pain which may radiate up to the jaw or ear, fever, tenderness, sore throat, easy fatigability, increased sweating, palpitations, tremors, heat intolerance, neck swelling, and general malaise, raised erythrocyte sedimentation rate (ESR), decreased thyroid-stimulating hormone (TSH) and less or no thyroid uptake [6,7]. In the acute period, due to the destruction of thyroid gland tissue, a large amount of thyroid hormone is released into the blood [7]. As a result, there can be features of hyperthyroidism such as tachycardia and increased metabolism. Then, SAT can burden and cause stress to the coronaries [8,9]. As there is depletion of the preformed thyroid hormone within few weeks, 30% of patients may progress into a hypothyroid phase. Fever of unknown origin (FUO) is defined as body temperature above 38.3°C (101°F) on three or more occasions and duration of illness of at least three weeks, in which no diagnosis was made after one week of hospital admission. Treatment ranges from medications such as nonsteroidal anti-inflammatory drugs (NSAIDs) to corticosteroids and thyroid hormone [1]. FUO as the sole presenting feature in subacute thyroiditis is very rare but reported in the literature [4,6,10]. Our patient presented as a case of fever which on workup was found to be secondary to SAT. The clinical response to the combination of NSAID and steroids in the patient further strengthens the diagnosis of SAT in our case.

## Case presentation

A 37-year-old male patient presented to the emergency department and got admitted to the ward with a history of fever with rigor and chills, swelling of neck and difficulty in swallowing for 25 days. He had tremors of hands and palpitation. There was no history of weight loss, cough, dysuria, joint pain, and skin rashes. There was no history of diabetes mellitus, hypertension, chronic obstructive airway disease (COPD), and dyslipidemia. On examination, he had a temperature of 100°F, tachycardia (pulse rate of 100 beats/min), and a small swelling was present on the anterior side of the neck. On investigations, blood culture was sterile, malaria, dengue, Widal test was negative, no pus cells in routine urine and no growth on culture, Mantoux test and viral markers were negative, RA factor was negative, chest x-ray and ultrasound abdomen were normal. Ultrasound of thyroid and thyroid scan was suggestive of thyroiditis. Other investigations are summarized in Tables 1, 2. He was managed with antibiotics, nonsteroidal anti-inflammatory drugs (NSAID), and glucocorticoids. Tab prednisolone was given in tapering doses (initially 10 mg twice daily for two weeks, reduced by 5 mg daily every two weeks). On follow-up post-discharge, his TSH increased to 43.42 ug/dL. The patient was prescribed levothyroxine tablet 100 mcg/day. Subsequently, his TSH normalized (Tables 1, 2).

**Table 1:** Investigations during hospital stay Hb: hemoglobin, TLC: total leukocyte count, CRP: C-reactive protein, ESR: erythrocyte sedimentation rate, ALT: alanine transaminase, AST: aspartate transaminase, ALP: alkaline phosphatase

Parameters	Values
Hb	11.8 gm/dL
TLC	11700/cumm
Platelets	182,000 per cumm
CRP	10.73 mg/dL
Serum Creatinine	0.98 mg/dL
Total Protein	7.9 gm/dL
ESR	32
Total Bilirubin	0.69
ALT	131.1 IU/L
AST	77.8 IU/L
ALP	130.1 IU/L

**Table 2:** Investigations on follow-up TSH: thyroid-stimulating hormone, T3: tri-iodothyronine, T4: tetra-iodothyronine, anti-TPO antibody: thyroid peroxidase antibody

Parameters	Upon admission	Post-discharge first follow-up	Next follow-up (after 2 months)
TSH (ug/dL)	0.02	43.42	3.7
T3 (ug/dL)	2.23	1.06	0.96
T4 (ug/dL)	18.15	4.2	9.84
Anti-TPO antibody (IU/mL)		1.6	

## Discussion

Fever of unknown origin (FUO) can be defined as a body temperature above 38.3°C (101°F) on three or more occasions and duration of illness of at least three weeks, in which no diagnosis was made after one week of hospital admission. FUO can be caused by infections, inflammations, malignancies, and rheumatologic disorders. In our case, the above etiology was excluded.

Fever (FUO), weakness, and fatigue can also be observed due to both inflammation and mild hyperthyroidism. Especially at the beginning period of SAT, palpitations, sweating, and tremors may occur due to high thyroid hormone levels in the blood. These findings usually disappear after four to 10 weeks, and the patient may develop asymptomatic, overt, or subclinical hypothyroidism.

SAT diagnostic criteria include pain in the neck and/or tenderness, raised ESR, absent or diminished thyroid uptake in the thyroid scan, and transient thyrotoxicosis at time of presentation in the absence of both excessive iodine intake history and history of medication which can be a cause of transient thyrotoxicosis (e.g., lithium, amiodarone, and various cytokines). Based on the natural history of the disease and the time of presentation, we can detect hypothyroidism or euthyroidism. In SAT, painless thyroid gland with fever as sole presentation is not very common. In this case, thyrotoxicosis as per laboratory results, decreased iodine uptake in thyroid scan, fever resolution with prednisolone and triphasic pattern of thyroid function in follow up points towards the diagnosis of SAT [9]. Self-limiting thyrotoxicosis of variable duration - which can last for a period of weeks or months - followed by hypothyroidism with final restoration to euthyroidism characterizes infection of the thyroid gland known as subacute thyroiditis [5]. Though the patient did not have tenderness in the neck swelling, it fulfills all the other criteria for SAT.

NSAIDs and/or steroids are the mainstays of therapy. Higher incidence of hypothyroidism after the treatment was found in the steroid therapy group [9]. An adequate guideline has not been established for the starting dose and duration of steroid. As per the symptom severity, 40 mg of prednisolone would be the usual starting dose for two weeks followed by gradual tapering by 5 mg over the next few weeks. In about 20-30% of the cases, patients seem to reveal overt or subclinical hypothyroidism with lower doses of steroids of 10 mg/day. After subacute thyroiditis, the chances of hypothyroidism can be 34% in six to 12 months and 15% after one year. Hypothyroidism can be treated with supplementation of levothyroxine with a stable clinical course as in our case. There is a need to follow-up till a euthyroid phase for several months in the course of subacute thyroiditis as thyroid dysfunction is common [9]. The clinical response to the combination of NSAID and steroids in the patient further strengthens the diagnosis of SAT in our case.

As per an randomized control trial (RCT) by Xu et al., in December 2020, to test the effectiveness and safety of 15 mg daily versus 30 mg daily of prednisolone as the initial dosage in patients with SAT to check non-inferiority effectiveness was found to have lower risk and more safety with ultimate patient benefit in long term. Oral corticosteroid therapy should be used in patients who do not respond to full doses of non-steroidal anti-inflammatory drugs (NSAIDs) [10].

## Conclusions

SAT is usually associated with a painful thyroid gland but can also uncommonly present as a painless thyroid gland and fever. In the primary care settings, SAT should be considered especially in young patients when no obvious cause for fever of unknown origin is found. A proper follow-up of the thyroid status is essential.

## References

[1] Ranganath R, Shaha MA, Xu B, Migliacci J, Ghossein R, Shaha AR (2016). de Quervain's thyroiditis: a review of experience with surgery. Am J Otolaryngol.

[2] Oláh R, Hajós P, Soós Z, Winkler G (2014). De Quervain thyroiditis. Corner points of the diagnosis. [Article in Hu]. Orv Hetil.

[3] Guven M (2020). Subacute thyroiditis in the course of coronavirus disease 2019: a case report. J Endocrinol Metab.

[4] Raj R, Yada S, Jacob A, Unnikrishnan D, Ghali W (2018). Fever of unknown origin as a sole presentation of subacute thyroiditis in an elderly patient: a case report with literature review. Case Rep Endocrinol.

[5] Alfadda AA, Sallam RM, Elawad GE, Aldhukair H, Alyahya MM (2014). Subacute thyroiditis: clinical presentation and long term outcome. Int J Endocrinol.

[6] Cappelli C, Pirola I, Gandossi E, Formenti AM, Agosti B, Castellano M (2014). Ultrasound findings of subacute thyroiditis: a single institution retrospective review. Acta Radiol.

[7] Liu R, Xu B, Luo H, Yang P, Xu BF, Xu G (2018). A case report of subacute thyroiditis and myocardial damage. Int J Clin Exp Med.

[8] Nakanishi A, Shunto J, Shunto R, Sata M, Bando H (2019). A case of subacute thyroiditis associated with complete occlusion of right coronary artery. J Diabetes Metab Disord Control.

[9] Brancatella A, Ricci D, Viola N, Sgrò D, Santini F, Latrofa F (2020). Subacute thyroiditis after SARS-COV-2 infection. J Clin Endocrinol Metab.

[10] Xu S, Jiang Y, Jia A (2020). Comparison of the therapeutic effects of 15 mg and 30 mg initial dosage of prednisolone daily in patients with subacute thyroiditis: protocol for a multicenter, randomized, open, parallel control study. Trials.

